# Immediate versus deferred percutaneous coronary intervention for patients with acute coronary syndrome: A meta-analysis of randomized controlled trials

**DOI:** 10.1371/journal.pone.0234655

**Published:** 2020-07-02

**Authors:** Weijun Li, Wenhua He, Yuqing Zhou, Yanfei Guo

**Affiliations:** Department of Cardiovascular Diseases of the Elderly, Chenzhou First People's Hospital, Chenzhou, China; Azienda Ospedaliero Universitaria Careggi, ITALY

## Abstract

Inconsistent results exist regarding the treatment effectiveness of immediate versus deferred percutaneous coronary intervention (PCI) in patients with acute coronary syndrome (ACS). This meta-analysis aimed to evaluate the efficacy and safety of immediate versus deferred PCI in ACS patients. PubMed, EMBASE, and Cochrane Library electronic databases were systematically searched from their inception up to August 2019. Random-effects models were employed to calculate pooled relative risks (RRs) and weight mean differences (WMDs) with 95% confidence intervals (CIs). A total of 10 randomized controlled trials (RCTs) that recruited 3350 patients were selected for inclusion in the final meta-analysis. Four trials included patients with non-ST elevation ACS (NSTEACS), whereas the remaining six trials included patients with ST elevation myocardial infarction (STEMI). There were no significant differences between immediate versus deferred PCI for the risk of major adverse cardiovascular events (NSTEACS patients: RR, 0.76, 95%CI, 0.33–1.75, P = 0.513; STEMI patients: RR, 1.24, 95%CI, 0.80–1.92, P = 0.335), myocardial infarction (NSTEACS patients: RR, 0.88, 95%CI, 0.27–2.81, P = 0.826; STEMI patients: RR, 0.86, 95%CI, 0.43–1.74, P = 0.678), all-cause mortality (NSTEACS patients: RR, 0.85, 95%CI, 0.38–1.88, P = 0.686; STEMI patients: RR, 1.16, 95%CI, 0.82–1.66, P = 0.407), target vessel revascularisation (NSTEACS patients: RR, 1.26, 95%CI, 0.29–5.43, P = 0.756; STEMI patients: RR, 1.01, 95%CI, 0.51–1.97, P = 0.988), or major bleeding (NSTEACS patients: RR, 0.99, 95%CI, 0.64–1.54, P = 0.972; STEMI patients: RR, 0.90, 95%CI, 0.45–1.77, P = 0.753). Although patients who underwent immediate PCI may experience increased incidences of cardiac death (RR, 1.19, 95%CI, 0.69–2.07, P = 0.525) and no or slow reflow (RR, 1.60, 95%CI, 0.91–2.84, P = 0.105), these increases were not statistically significant. We noted that immediate versus deferred PCI was associated with a reduced incidence of myocardial brush grade 3 (RR, 0.70, 95%CI, 0.56–0.88, P = 0.002); however, no significant differences were observed between immediate and deferred PCI for TIMI III flow (RR, 0.98, 95%CI, 0.93–1.03, P = 0.453), complete ST-segment resolution (RR, 0.93, 95%CI, 0.75–1.17, P = 0.548), and ejection fraction (WMD, −1.05, 95%CI, -2.58 to 0.49, P = 0.182). The findings of this study suggested that deferred PCI did not yield significant benefits for clinical endpoints. Further large-scale RCTs should be conducted to verify the findings of this study.

## Introduction

Acute coronary syndrome (ACS) includes unstable angina and myocardial infarction (MI) and is characterized by decreased perfusion of the heart muscles, which is associated with high risks of cardiovascular morbidity and mortality [1, 2]. Studies have illustrated baseline angiographic markers of disease burden, calcification, and lesion severity with significant predictive values for short- and long-term ischemic outcomes in ACS patients treated with early invasive strategies [3]. Currently, patients discharged after ACS treatment should be managed by lifestyle modification (e.g., physical activity planning, smoking cessation, and adherence to a healthy diet) and pharmacotherapy (e.g., acetylsalicylic acid, P2Y12-receptor inhibitors, beta-blockers, statins, angiotensin-converting enzyme inhibitors, and angiotensin receptor blockers) [4]. Although invasive strategies are widely introduced in combination with conservative treatment approaches, the treatment effectiveness and optimal timing of such approaches remain unclear [5–8].

Previous studies have illustrated differences between non-ST elevation ACS (NSTEACS) and ST elevation MI (STEMI) for ruptured plaques with a large necrotic core and abundant thrombus, with STEMI patients showing poor vascular healing processes and high risk of stent thrombosis [9–11]. Moreover, although the average daily ischemic risk and bleeding risk were similar within 1 year after percutaneous coronary intervention (PCI), we noted the incidence of average daily ischemic risk was higher than average daily bleeding risk within 2 weeks after PCI, especially for patients with STEMI or undergoing incomplete revascularization. Furthermore, the incidence of average daily ischemic risk was greater than average daily bleeding risk for patients with incomplete revascularization, while average daily bleeding risk was significantly higher than average daily ischemic risk for NSTEACS patients treated with ticagrelor [12]. Therefore, the risks of intervention for unstable plaque and ischemic events should be balanced with waiting for an invasive procedure. However, studies have reported inconsistent results regarding the effects of the use of early interventions on the risk of ischemic events and levels of cardiac injury biomarkers [13–15]. The current meta-analysis was conducted to evaluate the efficacy and safety of immediate versus deferred PCI in NSTEACS and STEMI patients on the basis of published randomized controlled trials (RCTs).

## Materials and methods

### Data sources, search strategy, and selection criteria

This study was conducted and reported according to the Preferred Reporting Items for Systematic Reviews and Meta-Analysis statement issued in 2009 [16]. Studies designed as RCTs and that investigated the treatment effectiveness of immediate versus deferred PCI in NSTEACS or STEMI patients were eligible for inclusion in this meta-analysis, with no restrictions placed on publication status or language. PubMed, EMBASE, and Cochrane Library electronic databases were systematically searched from their inception up to August 2019; we used the keywords “immediate” AND (“deferred” OR “delayed”) AND (“stenting” or “percutaneous coronary intervention” or “PCI” or “percutaneous coronary angioplasty”) AND (“acute coronary syndrome” or “myocardial infarction”) as the core search terms. Moreover, the website http://clinicaltrials.gov/ (US NIH) was reviewed for ongoing trials that registered their trial results but were not yet published. The reference lists of the collected articles were also manually reviewed to select any other RCTs that met the inclusion criteria.

The literature search and study selection were conducted by two authors following a standardized flow, and any conflicts were resolved by an additional author who read the original article. The inclusion criteria of this study included the following: (1) Patients: those diagnosed with NSTEACS or STEMI; (2) Intervention: immediate PCI; (3) Control: deferred PCI, which defined as the performed of PCI from within few hours up to 1 week and not restricted the timing of the second coronary angiogram; (4) Outcomes: the study should have reported at least one of following outcomes: major adverse cardiovascular events (MACEs), MI, all-cause mortality, target vessel revascularisation (TVR), major bleeding, cardiac death, no or slow reflow, myocardial brush grade 3 (MBG 3), TIMI III flow, complete ST-segment resolution, or ejection fraction level; and (5) Study design: RCT.

### Data collection and quality assessment

The collected data from individual trials included study, publication year, country, sample size, age, percentage of males, hypertension, diabetes mellitus, dyslipidemia, smoking, previous PCI, disease status, intervention, control, follow-up duration, and reported outcomes. The study quality was assessed using the Jadad scale, which was based on randomization, blinding, allocation concealment, withdrawals and dropouts, and the use of an intention-to-treat analysis [17]. The scoring system of the Jadad scale ranges from zero to five points with scores of four or more points considered to indicate high quality. Two authors conducted data collection and quality assessment, and the inconsistencies were settled by an additional author by referring to the original article.

### Statistical analysis

The treatment effectiveness of immediate versus deferred PCI for ACS patients was divided into categories and continuous data. The pooled relative risk (RR) and weighted mean difference (WMD) values with corresponding 95% confidence intervals (CIs) were calculated using a random-effects model, because of the true underlying effect varies across included trials were considered [18, 19]. Heterogeneity among included trials for each pooled outcome was assessed using *I*^*2*^ and Q statistics, and *I*^*2*^ > 50.0% or P < 0.10 was considered to indicate significant heterogeneity [20, 21]. Subgroup analyses were conducted based on disease status (NSTEACS or STEMI). Publication biases for investigated outcomes were calculated using funnel plots and Egger [22] and Begg [23] tests. All inspection levels for pooled effect estimates were two-sided, and P < 0.05 was considered statistically significant. STATA version 10.0 software (StataCorp, Texas, United States of America) was used for all statistical analyses.

## Results

### Literature search

**Fig 1:**
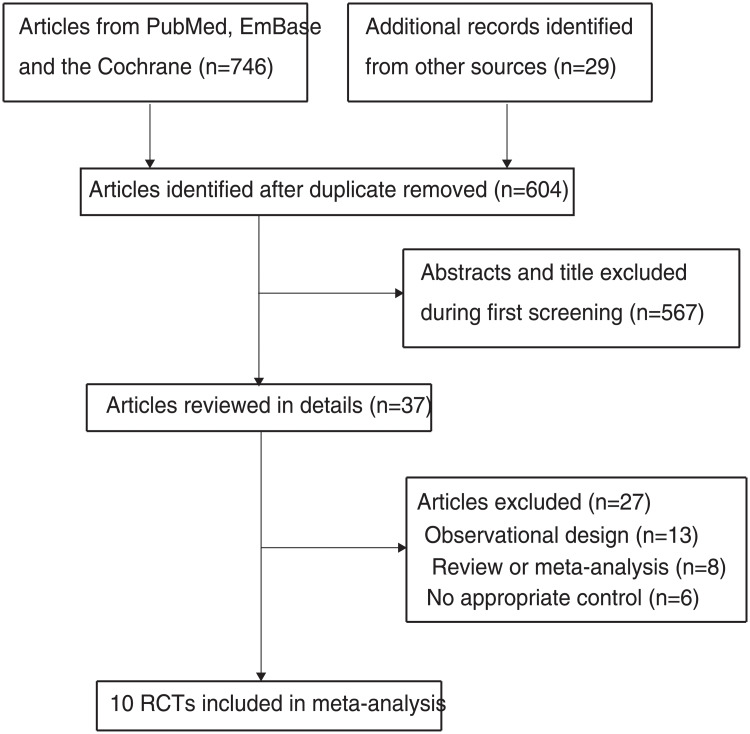
PRISMA flowchart for the study selection process.

The initial electronic search yielded 746 records, of which 142 were excluded for being duplicates. The titles and abstracts of the remaining 604 articles were reviewed, and 567 were further excluded because of irrelevant topics. A total of 37 studies were retrieved for further full-text evaluations, of which 27 studies were excluded because of an observational design (n = 13), being a review or meta-analysis (n = 8), or having no appropriate control groups (n = 6). The results of the manual reference list searches did not yield any other articles. Thus, a total of 10 RCTs were selected for final quantitative analysis (Fig 1) [14, 15, 24–31].

### Study characteristics

A total of 3350 patients from 10 RCTs were included in the final analysis. Four of these trials included 1219 patients with NSTEACS, whereas the remaining six trials included 2131 patients with STEMI. The baseline characteristics of the included studies and patients are summarized in Table 1. The duration of follow-up ranged from 30.0 days to 42.0 months, and 101–1215 patients were included in each trial. The mean age of the included patients ranged from 57.7 to 72.4 years, and the percentage of males ranged from 67.7% to 83.3%. Eight trials were conducted in Europe, one trial was conducted in the United States, and one trial was conducted in Korea. Seven of the included trials were of high quality, whereas the remaining three trials were of low quality.

**Table 1:** Baseline characteristics of the included studies.

Study	Country	Sample size	Age (years)	Percentage male (%)	Hypertension (%)	DM (%)	Dyslipidemia (%)	Smoking (%)	Previous PCI (%)	Disease status	Intervention	Control	Follow-up duration	Study quality
van’t Hof 2003 [14]	Netherlands	220	64.0	70.5	41.8	14.5	NA	34.5	14.5	Non-ST ACS	Immediate PCI	Deferred PCI (>12 h)	30 days	3
Riezebos 2009 [15]	Netherlands	142	62.5	71.8	43.7	19.7	35.2	38.7	23.2	Non-ST ACS	Immediate PCI	Deferred PCI (24–48 h)	30 days	4
Badings 2013 [24]	Netherlands	534	72.4	67.7	56.2	22.1	NA	23.8	19.5	Non-ST ACS	Immediate PCI	Deferred PCI (>48 h)	30 days	3
Milosevic 2016 [25]	Serbia	323	61.7	68.1	68.7	26.9	74.3	45.2	9.9	Non-ST MI	Immediate PCI (<2 h)	Deferred PCI (2–72 h)	30 days	4
Pasquale 2006 [26]	Italy	451	59.0	72.9	29.0	18.2	37.3	33.7	8.9	STEMI	Immediate PCI (<2 h)	Deferred PCI (12–72 h)	6 months	3
Carrick 2014 [27]	US	101	59.6	69.3	NA	12.9	NA	NA	4.0	STEMI	Immediate stenting	Deferred stenting (4–16 h)	6 months	5
Kim 2016 [28]	Korea	114	59.6	83.3	50.0	30.7	35.1	52.6	1.8	STEMI	Immediate stenting	Deferred stenting (3–7 days)	30 days	5
Kelbaek 2016 [29]	Denmark	1215	61.5	75.0	41.0	9.0	NA	52.5	NA	STEMI	Immediate stenting	Deferred stenting (>48 h)	42 months	5
Belle 2016 [30]	France	140	57.7	81.4	30.0	11.4	NA	67.1	4.3	STEMI	Immediate stenting	Deferred stenting (24–48 h)	6 months	5
Janssens 2019 [31]	Netherlands	110	61.7	70.9	33.6	9.1	27.3	46.4	6.4	STEMI	Immediate PCI	Deferred PCI (24–48 h)	12 months	4

### Major adverse cardiovascular events

**Fig 2:**
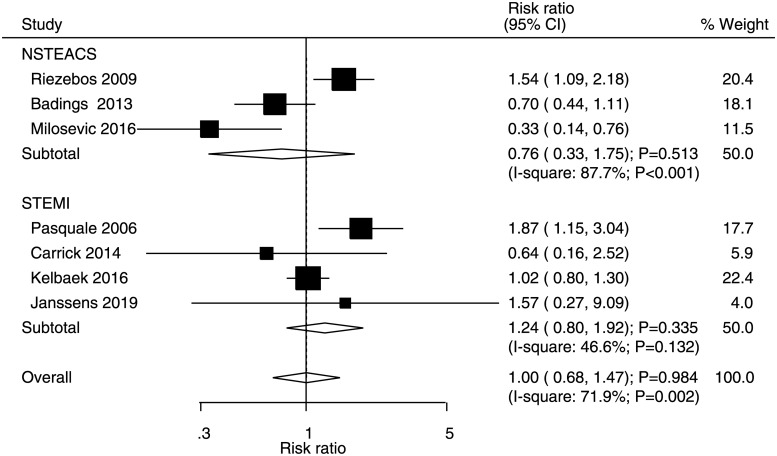
Immediate versus deferred PCI for major adverse cardiovascular events.

Data for the effects of immediate versus deferred PCI on the risk of MACEs were available in three and four studies of NSTEACS and STEMI patients, respectively. There were no significant differences between immediate and deferred PCI for the risk of MACEs in NSTEACS (RR: 0.76, 95% CI: 0.33–1.75; P = 0.513) and STEMI (RR: 1.24, 95% CI: 0.80–1.92; P = 0.335) patients (Fig 2). Significant heterogeneity was noted for MACEs in NSTEACS patients, whereas moderate heterogeneity was noted for MACEs in STEMI patients. No significant publication bias was detected for the risk of MACEs (P value for Egger = 0.598 and P value for Begg = 1.000; S1 Fig).

### Myocardial infarction

**Fig 3:**
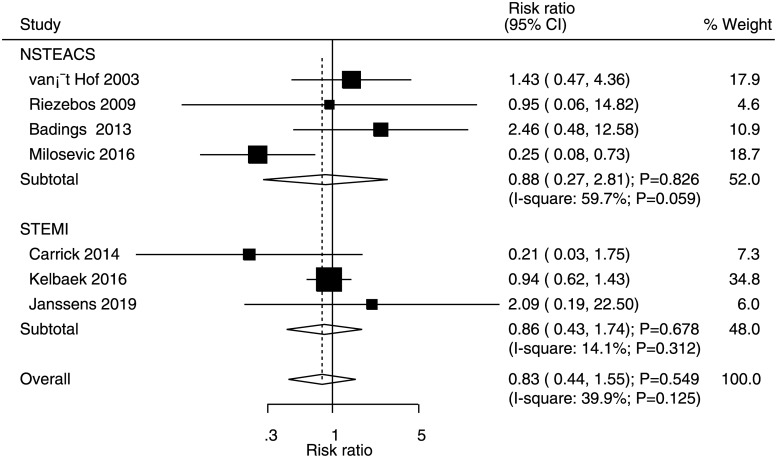
Immediate versus deferred PCI for myocardial infarction.

Data for the effects of immediate versus deferred PCI on the risk of MI were available in four and three studies of NSTEACS and STEMI patients, respectively. The summary RR indicated that immediate versus deferred PCI did not yield significant effects on the risk of MI in NSTEACS (RR: 0.88, 95% CI: 0.27–2.81; P = 0.826) and STEMI (RR: 0.86, 95% CI: 0.43–1.74; P = 0.678) patients (Fig 3). Significant heterogeneity was noted for MI in NSTEACS patients, whereas moderate heterogeneity was noted for MI in STEMI patients. No significant publication bias was observed for the risk of MI (P value for Egger = 0.892 and P value for Begg = 1.000; S2 Fig).

### All-cause mortality

**Fig 4:**
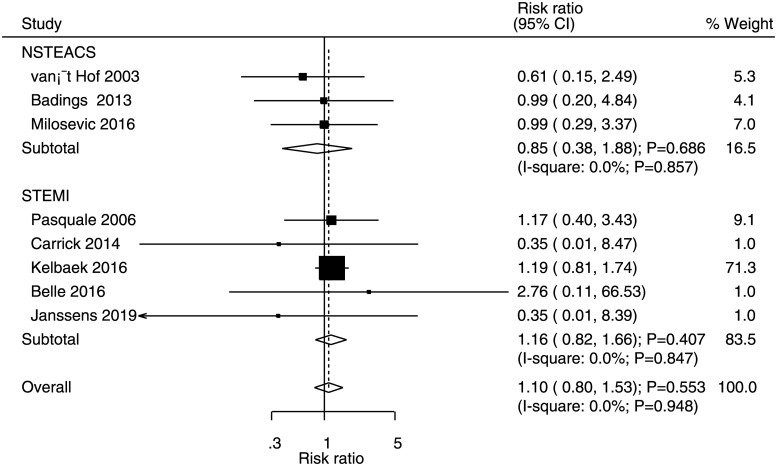
Immediate versus deferred PCI for all-cause mortality.

Data for the effects of immediate versus deferred PCI on the risk of all-cause mortality were available in three and five studies of NSTEACS and STEMI patients, respectively. No significant differences were noted between immediate and deferred PCI for the risk of all-cause mortality in NSTEACS (RR: 0.85, 95% CI: 0.38–1.88; P = 0.686) and STEMI (RR: 1.16, 95% CI: 0.82–1.66; P = 0.407) patients (Fig 4). There was no evidence of heterogeneity for all-cause mortality in NSTEACS and STEMI patients. No significant publication bias for the risk of all-cause mortality was observed (P value for Egger = 0.158 and P value for Begg = 0.386; S3 Fig).

### Target vessel revascularisation

**Fig 5:**
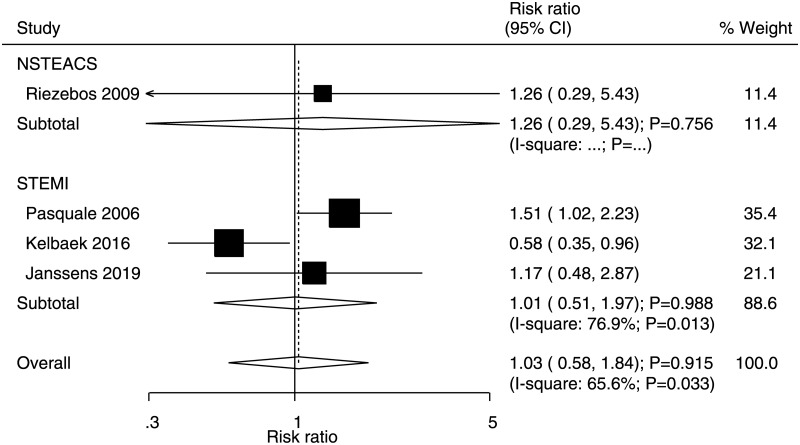
Immediate versus deferred PCI for TVR.

Data for the effects of immediate versus deferred PCI on the risk of TVR were available in one and three studies of NSTEACS and STEMI patients, respectively. There were no significant differences between immediate and deferred PCI for the risk of TVR in NSTEACS (RR: 1.26, 95% CI: 0.29–5.43; P = 0.756) and STEMI (RR: 1.01, 95% CI: 0.51–1.97; P = 0.988) patients (Fig 5). Moreover, significant heterogeneity was noted for TVR in STEMI patients. No significant publication bias was observed for TVR (P value for Egger = 0.900 and P value for Begg = 1.000; S4 Fig).

### Major bleeding

**Fig 6:**
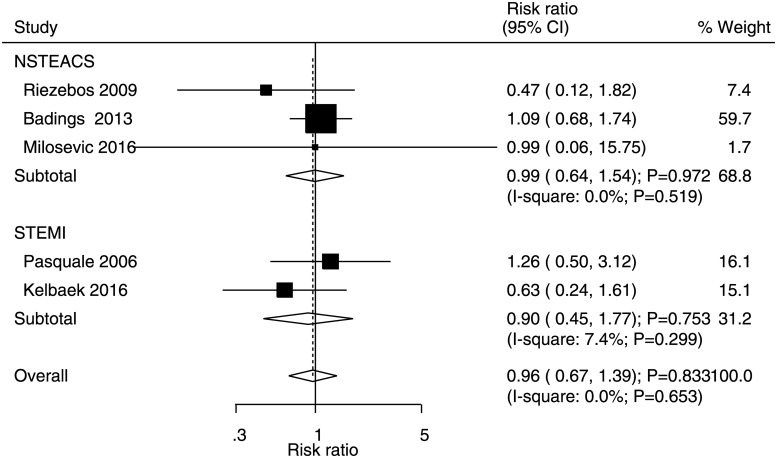
Immediate versus deferred PCI for major bleeding.

Data for the effects of immediate versus deferred PCI on the risk of major bleeding were available in three and two studies of NSTEACS and STEMI patients, respectively. Compared with deferred PCI, immediate PCI was not associated with a risk for major bleeding in NSTEACS (RR: 0.99, 95% CI: 0.64–1.54; P = 0.972) and STEMI (RR: 0.90, 95% CI: 0.45–1.77; P = 0.753) patients (Fig 6). No significant heterogeneity was detected for NSTEACS and STEMI patients. Moreover, no significant publication bias was observed for major bleeding (P value for Egger = 0.402 and P value for Begg = 0.221; S5 Fig).

### Cardiac death and no or slow reflow

**Fig 7:**
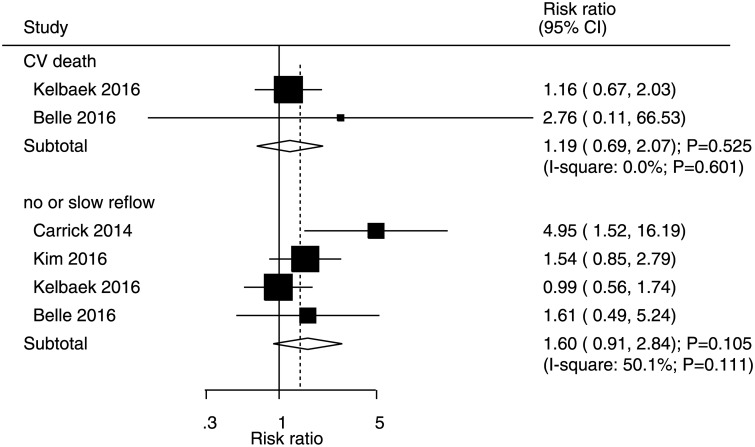
Immediate versus deferred PCI for cardiac death and no or slow reflow.

Data on the incidences of cardiac death and no or slow reflow in STEMI patients who underwent immediate PCI were available in two and four studies, respectively (Fig 7). The summary RRs indicated that immediate or deferred PCI was not associated with the incidences of cardiac death (RR: 1.19, 95% CI: 0.69–2.07; P = 0.525) and no or slow reflow (RR: 1.60, 95% CI: 0.91–2.84; P = 0.105). Furthermore, there was no significant heterogeneity for cardiac death, whereas potential significant heterogeneity for no or slow reflow was noted.

### Myocardial brush grade 3, TIMI III flow, and complete ST-segment resolution

**Fig 8:**
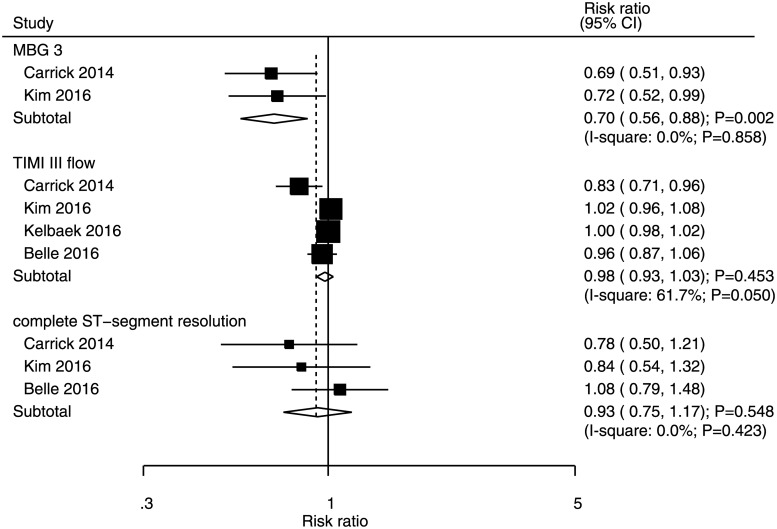
Immediate versus deferred PCI for MBG 3, TIMI III flow and complete ST-segment resolution.

Data on the incidences of MBG 3, TIMI III flow, and complete ST-segment resolution for STEMI patients who underwent immediate PCI were available in two, four, and three studies, respectively (Fig 8). Immediate versus deferred PCI was associated with a reduced incidence of MBG 3 (RR: 0.70, 95% CI: 0.56–0.88; P = 0.002); however, no significant differences between groups were noted for the incidences of TIMI III flow (RR: 0.98, 95% CI: 0.93–1.03; P = 0.453) and complete ST-segment resolution (RR: 0.93, 95% CI: 0.75–1.17; P = 0.548). No significant heterogeneity was detected for MBG 3 and complete ST-segment resolution, whereas significant heterogeneity was observed for TIMI III flow.

### Ejection fraction

**Fig 9:**
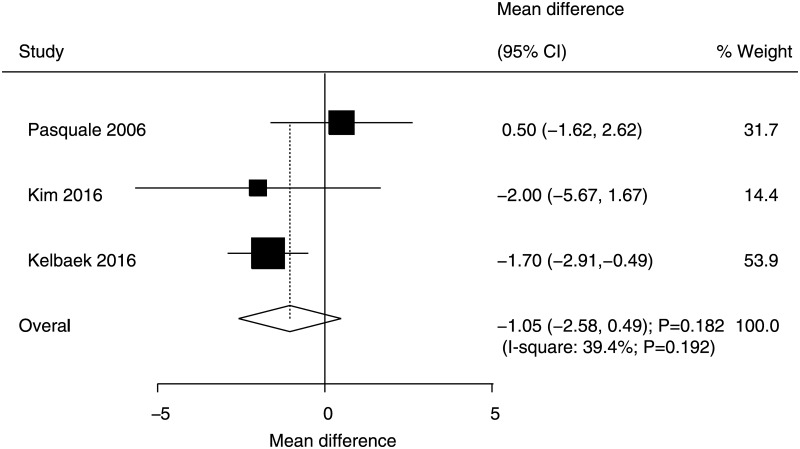
Immediate versus deferred PCI for ejection fraction.

Data for the effect of immediate versus deferred PCI on ejection fraction level were available in three trials. The pooled results suggested that no significant differences existed between immediate and deferred PCI for ejection fraction (WMD: −1.05, 95% CI: −2.58 to 0.49; P = 0.182), and no significant heterogeneity was detected across the included trials (Fig 9).

## Discussion

The current study provides updated results regarding immediate versus deferred PCI for STEMI patients and is the first study to pool the treatment effectiveness of immediate versus deferred PCI in NSTEACS patients. The current quantitative meta-analysis recruited 1219 NSTEACS patients and 2131 STEMI patients from 10 RCTs with a broad range of patient characteristics. The results of this study indicated that no significant differences existed between immediate and deferred PCI for the incidences of MACEs, MI, all-cause mortality, TVR, and major bleeding, irrespective of NSTEACS or STEMI status. Moreover, immediate or deferred PCI was not associated with cardiac death, no or slow reflow, TIMI III flow, complete ST-segment resolution, or ejection fraction in STEMI patients; however, compared with deferred PCI, immediate PCI was associated with a reduced incidence of MBG 3 in STEMI patients.

Several systematic reviews and meta-analyses have been conducted to evaluate the treatment effectiveness of immediate versus deferred PCI. Desch et al conducted a meta-analysis of nine RCTs and found that patients treated with immediate or early PCI showed reduction in all-cause mortality risk; however, the risk of stroke or major bleeding between groups was not statistically significantly different [32]. Freixa et al included five non-RCTs and one RCT in their study and found that delayed stent implantation may lead to beneficial angiographic outcomes in patients with acute MI [33]. Liu et al conducted a meta-analysis including 16 studies and found that STEMI patients treated with early PCI, as opposed to primary PCI alone, after fibrinolysis displayed similar results, which were better than those observed with ischemia-guided or delayed PCI [34]. A meta-analysis of three RCTs and seven non-RCTs suggested that deferred stenting provided beneficial outcomes for periprocedural composite events and abnormal flow in STEMI patients; however, no significant difference between immediate and deferred stenting for MACEs was noted [35]. Qiao et al conducted a meta-analysis including three RCTs and six non-RCTs and suggested that deferred stenting was associated with an improved left ventricular function; however, no significant differences between groups were noted in clinical endpoints [36]. Mahmoud et al conducted a meta-analysis including four RCTs and suggested that deferred stent implantation was associated with a lower incidence of no or slow reflow and improved MBG 3; however, association between the clinical endpoints in deferred and immediate stenting was not statistically significant [37]. Cassese et al conducted a meta-analysis including four RCTs and suggested that deferred stenting improves angiographic outcomes, but compared with immediate stenting, deferred stenting has no significant effects on imaging or clinical outcomes [38]. However, the above studies included smaller number of RCTs and the results were not stable. Moreover, whether the treatment effectiveness of immediate versus deferred PCI differs in NSTEACS patients remains unclear. Therefore, the current meta-analysis was conducted to evaluate the efficacy and safety of immediate versus deferred PCI for NSTEACS and STEMI patients.

The summary results indicated that immediate versus deferred PCI did not yield any significant effects on MACEs, MI, all-cause mortality, TVR, and major bleeding, irrespective of NSTEACS or STEMI status. Although no significant differences between groups were noted for cardiac death, no or slow reflow, TIMI III flow, complete ST-segment resolution, and ejection fraction, we noted that deferred PCI may have a beneficial effect on MBG 3. Several reasons could help interpret the above results: (1) deferred PCI allows for a better sizing of the lesion and artery, which is associated with optimized stent selection [39]; (2) patients treated with deferred PCI require better evaluations of the revascularization strategy, which could avoid unnecessary stenting for nonsignificant residual stenosis [40]; and (3) deferred PCI strategy always involves repeated angiograms, which could detect nonculprit arteries in patients with multivessel lesions [41]. However, smaller number of included trials could be the reason behind no significant differences between immediate and deferred PCI for the incidences of clinical endpoints, and the power may not have been sufficient to detect potential differences between immediate and deferred PCI. Moreover, treatment with deferred PCI strategy is associated with higher costs, prolonged hospitalization, and high risk of reocclusion.

Although the results of our study did not reveal any significant differences between immediate and deferred PCI, our findings suggested that deferred PCI is safe for the treatment of NSTEACS and STEMI patients. However, several limitations of this study should be mentioned: (1) variations in disease status and background therapies could affect the prognosis of NSTEACS and STEMI patients; (2) several investigated outcomes were reported in few trials, and the results regarding immediate versus deferred PCI were variable; and (3) this meta-analysis was based on published study levels; thus, publication bias was inevitable and detailed analysis results were not obtained.

## Conclusion

The results of this study indicated that compared with deferred PCI, immediate PCI may be associated with poor MBG 3 outcomes; however, no significant differences between the groups were noted for other clinical endpoints. Our results are stable and were not affected by the inclusion of NSTEACS or STEMI patients. The results of this study should be verified by further large-scale RCTs in accordance with patients’ characteristics.

## Supporting information

S1 Fig Funnel plot for major adverse cardiovascular events. (TIF)

S2 Fig Funnel plot for myocardial infarction. (TIF)

S3 Fig Funnel plot for all-cause mortality. (TIF)

S4 Fig Funnel plot for TVR. (TIF)

S5 Fig Funnel plot for major bleeding. (TIF)

S1 Checklist PRISMA 2009 checklist. (DOC)
