# Supplementary figures and images for: Immediate versus deferred percutaneous coronary intervention for patients with acute coronary syndrome: A meta-analysis of randomized controlled trials
Source: PLoS One. 2020 Jul 2;15(7):e0234655. doi: 10.1371/journal.pone.0234655 (PMC7332029; doi:10.1371/journal.pone.0234655)

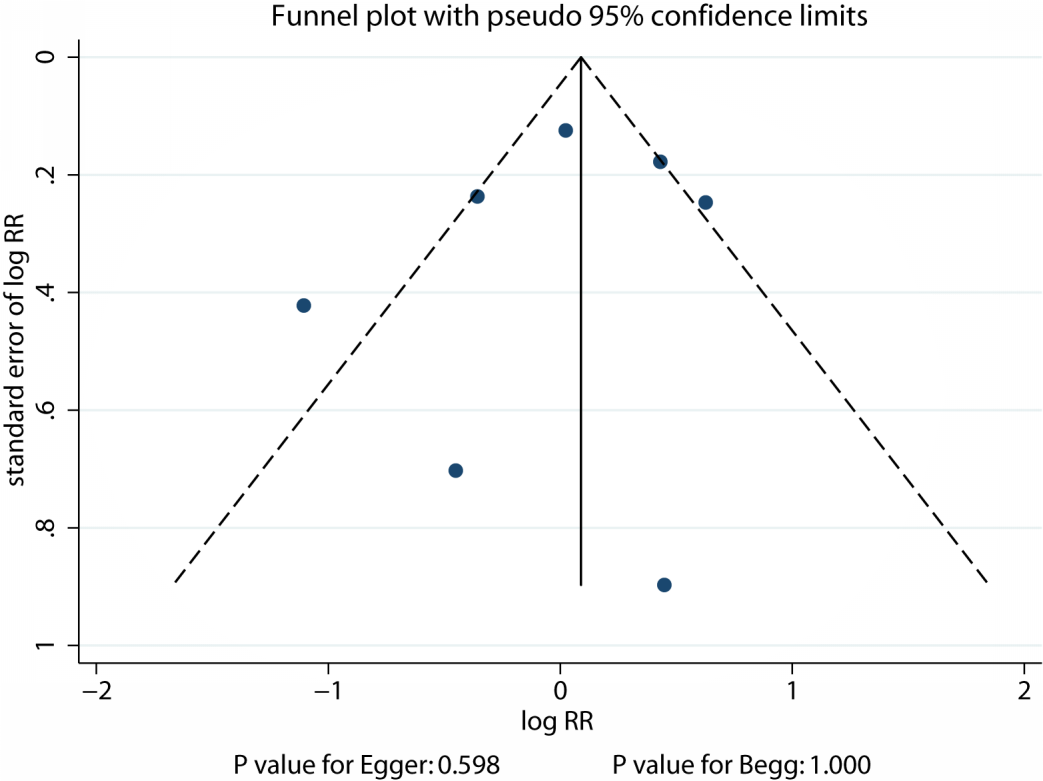

Supplement: S1 Fig — (TIF) [file pone.0234655.s001.tif]

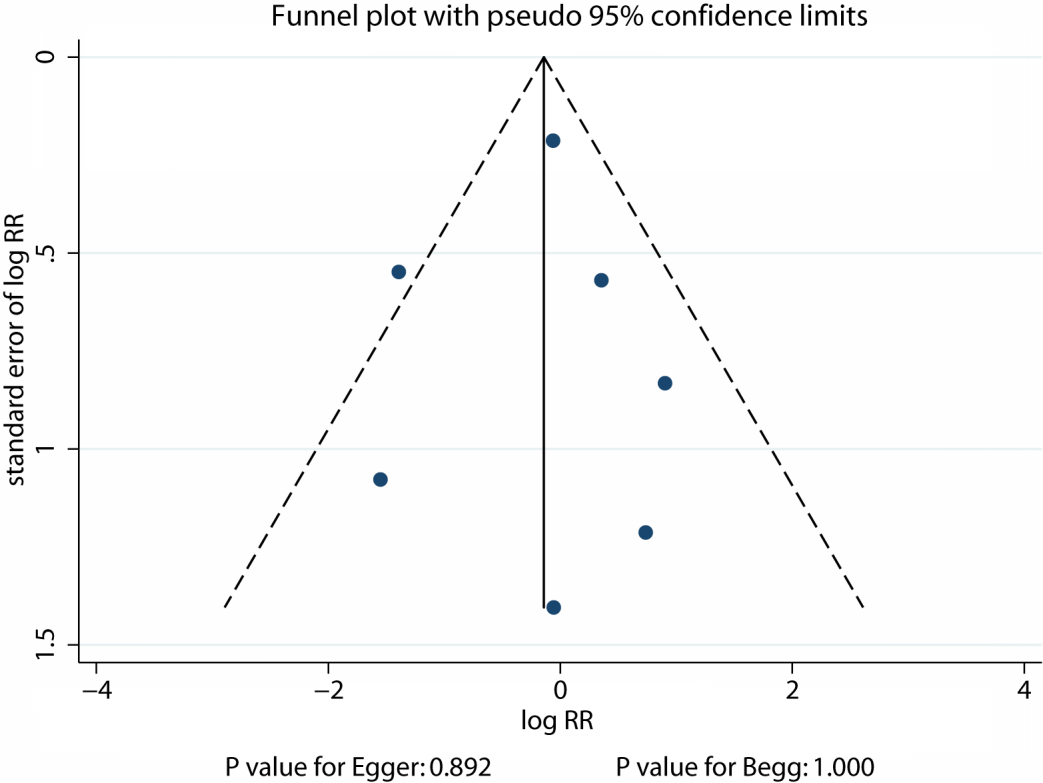

Supplement: S2 Fig — (TIF) [file pone.0234655.s002.tif]

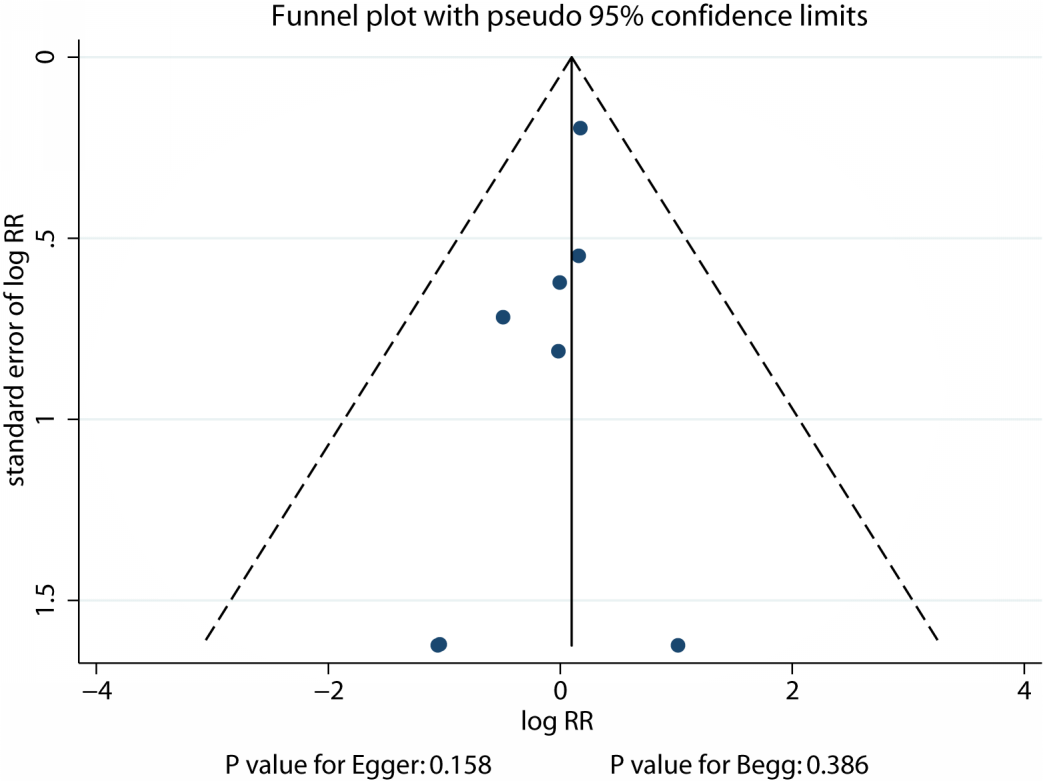

Supplement: S3 Fig — (TIF) [file pone.0234655.s003.tif]

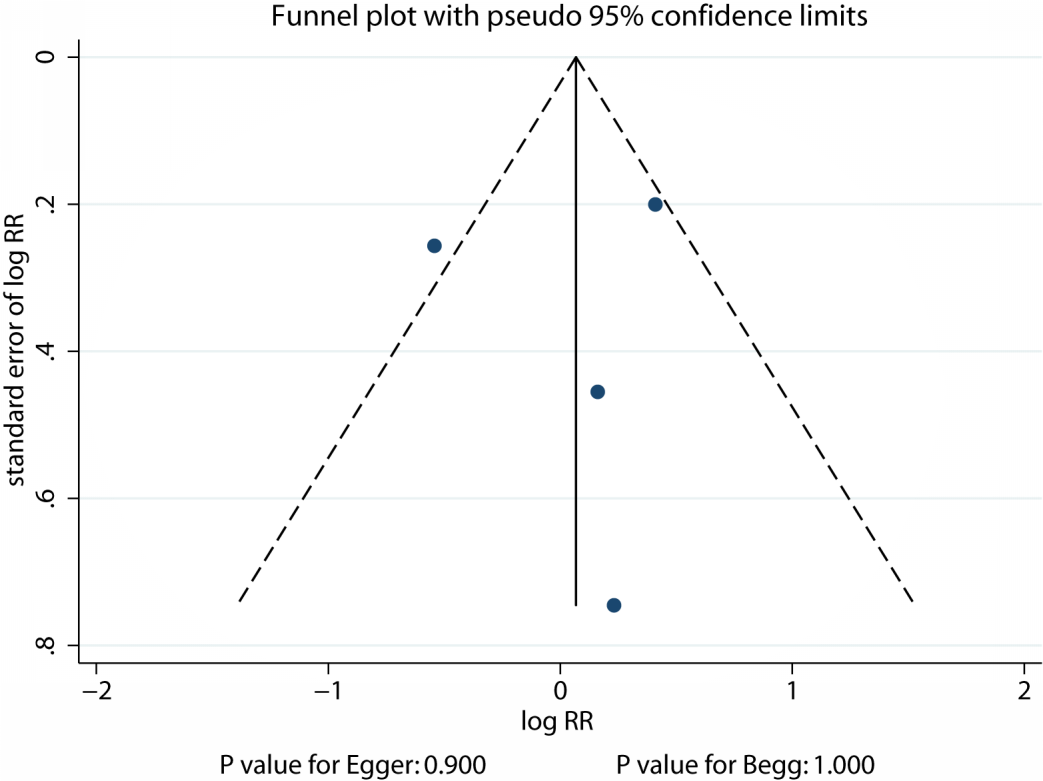

Supplement: S4 Fig — (TIF) [file pone.0234655.s004.tif]

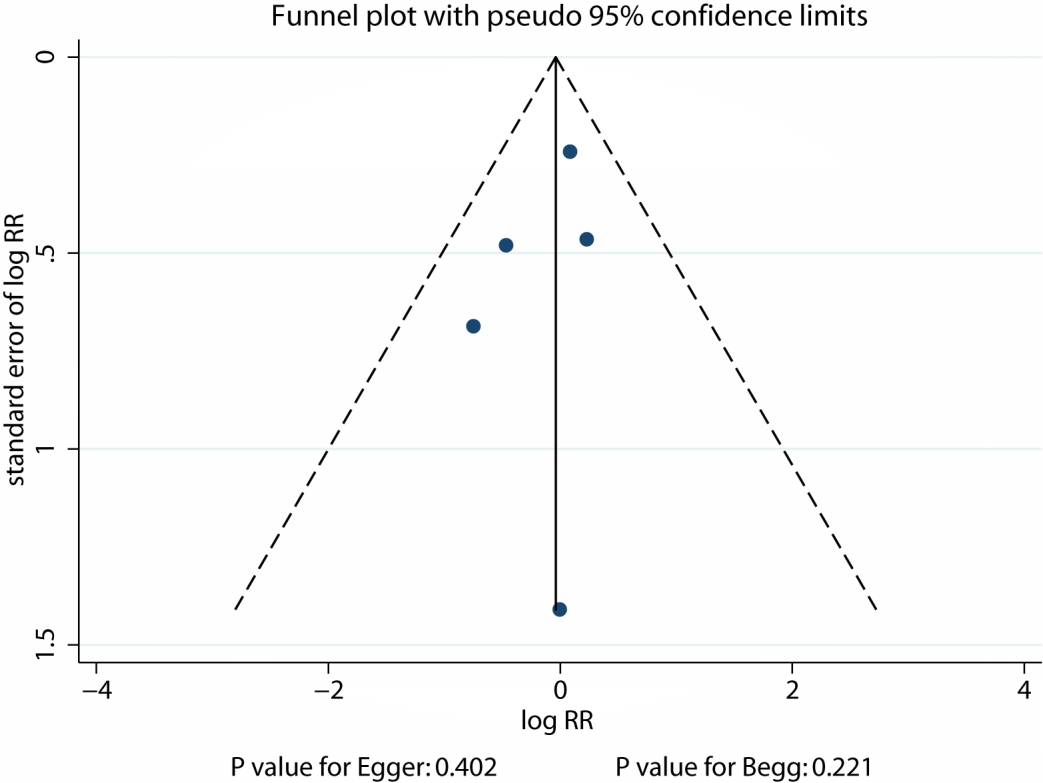

Supplement: S5 Fig — (TIF) [file pone.0234655.s005.tif]
